# Versatile use of rtTA-expressing retroviruses in the study of neurodegeneration

**DOI:** 10.18632/oncotarget.14386

**Published:** 2016-12-30

**Authors:** Catia M. Teixeira, Jesús Ávila, María Llorens-Martín

**Affiliations:** Department of Molecular Biology, Centro de Biología Molecular “Severo Ochoa” (CBMSOCSIC-UAM), Universidad Autónoma de Madrid, Madrid, Spain

**Keywords:** adult neurogenesis, tTA, retrovirus, GSK-3β, physical exercise, Neuroscience

The tetracycline-regulated transactivator (tTA) was used in combination with the *E.coli tet* operon (TetR) to induce the expression of target genes both *in vivo* and *in vitro* [1]. In this system, the activation of TetR occurs only after the binding of the tTA element. Thus, target genes will be overexpressed only in those cells in which TetR and tTA are co-expressed. Tissue or cell specificity depends on the promoter that drives the expression of tTA. In addition, the temporal control of gene expression is regulated by the presence of Tetracycline (or, more commonly, its analogue, Doxycycline, which crosses the blood-brain barrier easily). This molecule triggers a conformational change in tTA (natively active) that prevents its binding to TetR and suppresses transcription (tet-OFF system). More recently, variations in this system include the use of the reverse tetracycline-regulated transactivator (rtTA), which requires the presence of tetracycline to bind to tetR (tet-ON system).

Numerous combinations of mice engineered by tTA/TetR systems have been generated in recent decades. In this regard, a model of particular relevance in the study of Alzheimer disease (AD) is the GSK-3β overexpressing (GSK-3β-OE) mouse [2]. This line is obtained by crossing tetR-GSK-3β mice (which carry a bi-directional TetR promoter followed by a GSK-3β cDNA in one direction and a cDNA encoding β-Galactosidase (β-Gal) fused to a nuclear localization signal in the other) with CamKII/tTA mice. Thus, in the resulting GSK-3β-OE mice, this kinase is overexpressed in hippocampal and cortical neurons [3]. These mice exhibit increased neuronal Tau phosphorylation. Although these animals mimic several features of the AD brain scenario, the overexpression of a pathogenic protein in a general population of cells unavoidably triggers the activation of a series of non-cell-autonomous consequences, such as neuroinflammation and microglial activation. These phenomena may act as a confounding factor and may hinder unambiguous conclusions regarding the specific contribution of a given protein to the neurodegenerative process.

**Figure 1:**
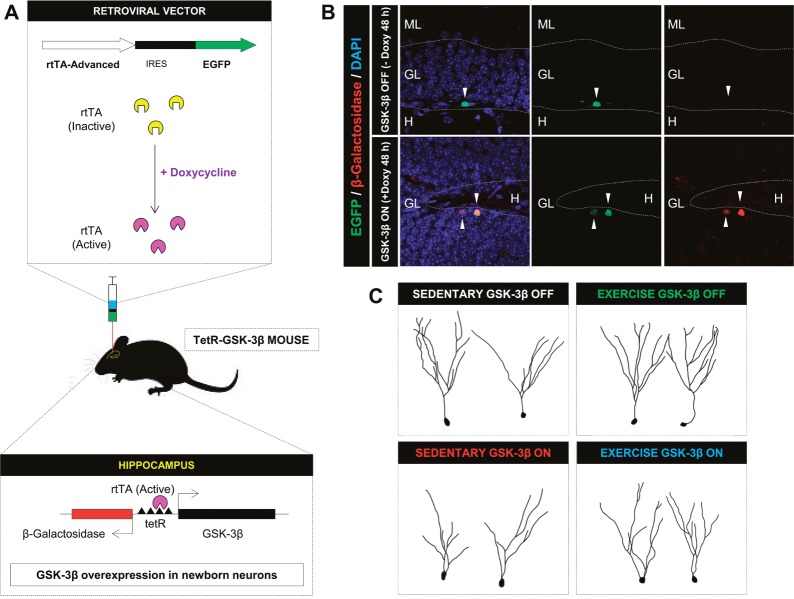
The *in vivo* use of rtTA-expressing retroviruses We have used an rtTA-IRES-GFP-expressing retrovirus **(A)** to deliver the rtTA element into the dividing newborn granule neurons of tetR-GSK-3β mice. After the binding of the tTA element to the TetR in the cells infected by the retroviruses, and in the presence of Doxycycline, GSK-3β will be selectively overexpressed in the target cells **(B)**. By using this system we have obtained a rapid induction of GSK-3β overexpression in these cells **(C)**. Furthermore, by combining this system with four weeks of voluntary exercise, we observed a markedly decreased (or even absent) cellular response to the stimulatory actions of physical exercise **(D)**.

In order to avoid these indirect effects, and taking advantage of the fact that tTA can be delivered to target cells by viral vectors, we have recently developed a novel strategy, which, to the best of our knowledge, has not been applied to the study of neurodegenerative processes in the CNS. This strategy is based on the use of tetR-GSK-3β mice and the hippocampal delivery of rtTA by means of an rtTA-IRES-GFP-expressing retrovirus (Figure 1A). Using this system, we achieved rapid and selective *in vivo* overexpression of GSK-3β in the hippocampal neurons infected by the retrovirus (Figure 1B).

The *in vivo* use of retroviral vectors in the CNS became generalized after the seminal work by F.H. Gage and collaborators [4]. These authors demonstrated the resulting infection of dividing newborn granule neurons, a cell population subjected to continuous neurogenesis throughout life. Adult hippocampal neurogenesis (AHN) is severely damaged in AD patients [5]. In this regard, we showed that the injection of an rtTA-expressing retrovirus into the hippocampus of tetR-GSK-3β mice causes a dramatic cell-autonomous alteration of newborn granule neuron morphology and connectivity. It should be noted that GSK-3β overexpression can be monitored by means of two reporter proteins, namely EGFP (encoded by the retroviral genome) and β-Gal (encoded by murine genome). In addition, we detected an increase in the levels of Tau phosphorylation in EGFP^+^/β-Gal^+^ cells [6]. In addition, by combining this system with four weeks of voluntary exercise, we observed a markedly decreased (or even absent) cellular response to the stimulatory actions of physical exercise (C). These data further confirm the pivotal role played by GSK-3β in regulating not only the physiological maturation of newborn granule neurons but also their response to external stimuli.

In summary, the delivery of tTA by means of viral vectors has three major advantages with respect to classical murine lines. First, it is straightforward to generate viral constructs in which the expression of the tTA element is driven by specific promoters; second, the use of different types of viral vector (either retro- lenti- or adeno-associated- viruses) allows higher flexibility in terms of the cellular populations infected; and, last but not least, the use of this type of vector (especially in the case of retroviruses) almost completely removes non-cell-autonomous effects derived from the expression of pathologic proteins in a wide range of cell populations.

Hence, we propose that the use of viral vectors expressing either tTA or rtTA elements in combination with tetR mouse models will contribute to improving our knowledge of the cell-autonomous effects of proteins thought to be pathological in certain contexts. In addition, these data may contribute to the design successful treatments for, but not limited to, neurodegenerative disorders.
